# The Effect of Fremanezumab on Pain in Patients with Complex Regional Pain Syndrome: Study Protocol of a Randomized, Double-Blind, Proof-of-Concept, Placebo-Controlled Trial

**DOI:** 10.3390/brainsci15050468

**Published:** 2025-04-28

**Authors:** Abarajitha Thiyagarajah, Astrid Juhl Terkelsen, Frank Birklein, Nanna Brix Finnerup, Sandra Sif Gylfadottir

**Affiliations:** 1 Danish Pain Research Center, Department of Clinical Medicine, Aarhus University, 8200 Aarhus N, Denmark; finnerup@clin.au.dk (N.B.F.); sifgyl@clin.au.dk (S.S.G.); 2 Department of Clinical Medicine, Aarhus University, 8200 Aarhus N, Denmark; astrid.terkelsen@clin.au.dk; 3 Department of Neurology, Aarhus University Hospital, 8200 Aarhus N, Denmark; 4 Department of Neurology, University Medical Centre Mainz, 55131 Mainz, Germany; frank.birklein@unimedizin-mainz.de

**Keywords:** complex regional pain syndrome, randomized controlled trial, calcitonin gene-related peptide, monoclonal antibodies, pain, numeric rating scale, chronic primary pain

## Abstract

**Background/Objectives:** Complex regional pain syndrome (CRPS) is a primary pain condition that can develop in a limb after a trauma. Although the condition is rare, it may cause lifelong pain and disability. Evidence-based treatments are limited. Neurogenic inflammation induced by the release of neuropeptides, such as calcitonin gene-related peptide (CGRP), is thought to play an important role in the pathophysiology of CRPS. Recently, drugs targeting CGRP have proven to be effective and well tolerated in the treatment of migraine, but their efficacy in other pain conditions, including CRPS, is unclear. The aim of this study is to assess the efficacy of the anti-CGRP antibody fremanezumab on pain in CRPS. **Methods:** In this randomized, double-blind, placebo-controlled, proof-of-concept study, 60 adult patients with CRPS with a disease duration of 3–36 months are randomized to treatment for eight weeks with fremanezumab 225 mg or placebo administered subcutaneously at a 1:1 rate. The primary objective is to compare the change in pain intensity from baseline to the last week of treatment between fremanezumab and the placebo. Other objectives are to assess pain relief and differences in clinical signs between the groups and to examine if the effect can be predicted by CGRP biomarkers. Adverse events and blinding will also be assessed. **Conclusions:** If found effective, fremanezumab and other anti-CGRP antibodies may emerge as a mechanism-based treatment option for patients with CRPS, which could hopefully improve the overall care of patients with this devastating disease.

## 1. Introduction

Complex regional pain syndrome (CRPS) is a chronic pain condition that can occur distally in a limb following trauma or surgery. The pain is unproportionate in intensity and duration to the usual course following a similar trauma or surgery. Besides ongoing pain, the condition is characterized by sensory changes (e.g., allodynia and hyperesthesia); changes in temperature, skin color, and sweating; edema; and motor and trophic disturbances in the affected limb [1,2]. Although CRPS is an uncommon disorder, it comes with a great personal and economic burden. Indeed, among CRPS patients with a symptom duration of at least one year, pain and motor impairment are prevalent symptoms, and up to 40% of patients are unable to return to work [3]. Furthermore, psychological distress is reported more commonly among CRPS patients [4], and CRPS is associated with increased health care costs [5,6].

The etiology of CRPS is thought to be multifactorial. One of the underlying mechanisms believed to play a significant role in the pathophysiology of CRPS is neurogenic inflammation. Following the initial limb trauma, post-traumatic inflammation is seen with release of inflammatory mediators, which is thought to induce the activation of peripheral nociceptors. Besides causing pain, the activation of peptidergic nociceptors results in the release of neuropeptides such as calcitonin gene-related peptide (CGRP) and substance P [7,8]. This is believed to induce neurogenic inflammation, leading to edema, increased blood flow, and the recruitment of inflammatory cells [9,10,11]. This subsequently results in the release of cytokines, inducing further activation and sensitization of peptidergic nociceptors [12]. Thus, augmented neurogenic inflammation could explain some of the key features of CRPS, e.g., pain, swelling, heat, and redness.

Despite considerable progress in understanding the pathophysiology of CRPS, treating it is challenging. Evidence-based treatments are limited [13]. In the absence of treatment options, current treatment recommendations for pain in CRPS are the same as for neuropathic pain [14,15].

Recently, drugs targeting CGRP have become available and have been shown to be effective in the treatment of migraine [16,17]. However, the role of these anti-CGRP antibodies in other pain conditions is sparsely elucidated. A recently conducted randomized controlled trial on the efficacy of erenumab, an anti-CGRP antibody, on trigeminal neuralgia showed no effect on pain [18]. In a small retrospective study of patients with both migraine and neuropathic pain receiving treatment with anti-CGRP antibodies for their migraine, the patients reported a 41.7% reduction in their neuropathic pain [19]. While the role of CGRP in neuropathic pain is uncertain, the role of CGRP in CRPS is thought to be more crucial. Indeed, preclinical and clinical studies suggest that CGRP mediates pain and inflammation in CRPS [11,20,21,22,23,24]. This makes CGRP a possible target for a mechanism-based treatment approach to CRPS. To the authors’ knowledge, no prior studies have investigated the efficacy of anti-CGRP antibodies on pain in patients with CRPS.

The main aim of this study is to investigate the efficacy of fremanezumab, a drug targeting CGRP, on pain in patients with CRPS. The secondary aims are to assess pain relief and differences in clinical signs between the groups and to explore if the effect can be predicted by CGRP biomarkers.

## 2. Materials and Methods

### 2.1. Study Design and Settings

**Figure 1:**
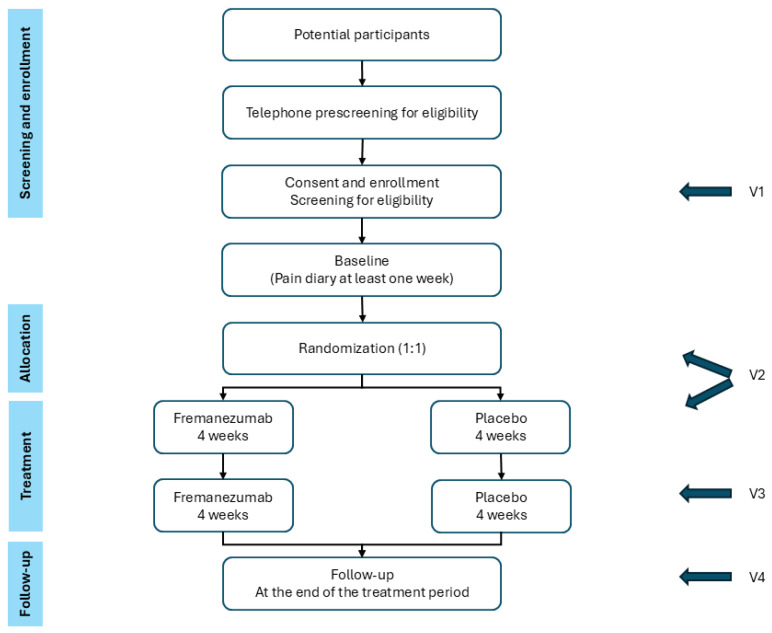
Flow of participants. Blue arrows denote visits. V: visit.

The study is designed as an investigator-initiated randomized, double-blind, placebo-controlled, proof-of-concept single-center study with two parallel groups. It is being conducted at the Danish Pain Research Center (DPRC), Department of Clinical Medicine and Department of Neurology, Aarhus University and Aarhus University Hospital, Denmark. The study plans to include 60 patients. Patients are randomized to one of two arms: fremanezumab 225 mg or placebo (isotonic saline) administered subcutaneously at a 1:1 rate. The study comprises a baseline period of one week and a treatment period of eight weeks (Figure 1). Outcomes are evaluated at the end of the eighth week.

### 2.2. Recruitment of Participants

The patients are recruited from the outpatient pain clinic at the Department of Neurology at Aarhus University Hospital, other pain clinics and hospital departments in Denmark, and through advertising. The written patient information is forwarded to the interested patients, and a telephone interview is conducted. During the telephone contacts, the patients are prescreened for eligibility, and information is given about the trial and what participation in the trial implies for the patients. If the patients are deemed eligible following the prescreening, they are invited to a screening visit at the DPRC, where written informed consent is obtained.

### 2.3. Study Visits

All visits are planned at the beginning of the study. Visit 2 is defined as day 1. All study visit days are calculated from this visit date (Table 1).

At visit 1 (screening visit), the patient sits down with a member of the study team, discusses any additional questions, and provides informed consent. After informed consent is obtained, the patient is further assessed for study eligibility. Previous and current medical histories are obtained, and focused neurological and physical examinations are performed to establish or confirm the CRPS diagnosis, and for fertile women, a pregnancy test is taken. The patients are trained to report pain intensity.

Registration in the pain diary starts in the baseline period. If visit 1 is scheduled for more than seven days before visit 2, the patients are instructed to fill out the pain diary for the last seven days minimum preceding visit 2. The patients should have an average daily pain (ADP) intensity of at least 4 and not above 9 on a numeric rating scale (NRS) from 0 to 10 (0 = no pain; 10 = worst possible pain) in the baseline period. This is not revealed to the patient.

At visit 2, the pain diary is used to confirm the inclusion and exclusion criteria (Table 2). Patients are, thereafter, randomized to treatment with fremanezumab or placebo. They receive the first dose of the study drug, and outcomes are accessed (see section on outcomes). Registration in the pain diary continues until the last week of treatment (week 8).

At visit 3, after four weeks of treatment, patients receive the second dose of treatment. An assessment of concomitant medication and any potential side effects is conducted, and blood pressure and pulse are measured. Additionally, a questionnaire on pain relief is filled in. Patients will not receive the second dose of study medicine if the side effects are unacceptable or if they wish to stop for other causes, but all patients who have taken at least one of the two doses of study drug or placebo are asked to continue to participate in the study as described below.

At visit 4, after the treatment period of eight weeks, outcomes are assessed again. An assessment of concomitant medication and any potential side effects and completion of the study is also conducted.

During the treatment period, the patients are contacted by telephone (at least twice and more often if necessary) and interviewed in a standardized manner to ensure compliance and to collect data on safety, side effects, and concomitant medication. Additional contact may be arranged if necessary.

### 2.4. Study Procedures

The study procedures are defined as follows:

1. Pain diary: Patients fill in a pain diary, where they report the mean ADP and the least and worst pain in the previous 24 h. It is preferred that the patients complete the diary electronically via REDCap (Research Electronic Data Capture) hosted at Aarhus University [28,29]; otherwise, they are provided with a paper diary.
2. Demographics: Interview questions include those regarding age, sex, race, ethnicity, education, and occupational status. Questions regarding limb domain before CRPS and CRPS duration are also asked.
3. Blood samples: We perform a measurement of inflammatory markers in serum. A total of 10 mL blood is drawn at visits 2 and 4, and the specimen is refrigerated and stored in a biobank at Aarhus University Hospital.
4. Skin biopsy: Biopsy is performed following published guidelines from the most painful area on the dorsal site of the affected limb and corresponding limb (3 biopsies) [30]. After intradermal injection of 1% lidocaine, a skin biopsy is obtained using sterile technique with a disposable 3 mm punch. Samples are fixed with Zamboni fixative overnight and then cryoprotected in 20% sucrose 0.1 M phosphate buffer. The biopsy specimen is refrigerated and stored in a biobank at Aarhus University Hospital. CGRP and other markers of inflammation are measured and quantified in skin biopsies using stereology [31].
5. Quantitative sensory testing (QST): QST is a psychophysical method used to examine the function of the sensory nervous system using standardized thermal and mechanical stimuli, as proposed by the German Research Network on Neuropathic Pain. We measure the cold and warm detection thresholds (CDT and WDT), cold and heat pain thresholds (CPT and HPT), dynamic mechanical allodynia (DMA), mechanical detection and pain thresholds (MDT and MPT), and wind-up ratio (WUR), performed at the most painful area and at a corresponding non-painful area on the contralateral limb [32].
6. Outcomes: See Section 2.7 on outcomes.

### 2.5. Randomization and Blinding

Randomization is carried out at a 1:1 rate, stratified by sex, via computer-generated randomization in REDCap using block sizes unknown to the investigators. Randomization is carried out after the baseline period if the patients meet the criteria of at least 4 on an NRS from 0 to 10.

It is not possible to make the active drug and placebo syringes identical in appearance, and they are, therefore, prepared and administered by two unblinded third-party personnel (to ensure double control) at the study site. The active substance comes in a package from the manufacturer, and the information on the package is not covered by a research label. The placebo (isotonic saline) is prepared on the study site in a separate room from where the subject receives the treatment. The needles are identical, and the subject is blindfolded during the injection. Hence, the study is double-blind (patients, health care providers, and investigators/study personnel). The sequence of the randomization numbers is only available to the unblinded personnel. The code for randomization is stored in a locked room until the study is completed. For each randomization code, there is a sealed envelope with information on the treatment given. The code envelope is only unsealed/opened if continued treatment requires knowledge of the randomization code.

### 2.6. Intervention

Fremanezumab 225 mg or placebo (isotonic saline) is given via subcutaneous injection every four weeks: in total, twice during the treatment period of eight weeks (at visits 2 and 3). The injections are administered on the study site by unblinded study personnel (see Section 2.5 on randomization and blinding).

Due to the risk of hypersensitivity reactions, the participants are monitored at the study site at least 30 min after the administration of the study drug.

### 2.7. Outcomes

Primary outcomes are as follows:

1. Difference between groups (placebo vs. active treatment) in the change in self-reported daily ratings of mean ADP intensity in the affected limb from the baseline week (week −1) to the last week of treatment (week 8), as experienced during the past 24 h, rated on an 11-point NRS from 0–10 (0 = no pain; 10 = worst possible pain): the primary outcome is validated and recommended as a first Core Outcome Measurement set for complex regional PAin syndrome Clinical sTudies (COMPACT) and a standard measure of chronic pain in clinical trials [33,34,35,36].

Secondary outcomes are as follows:

1. Difference in pain relief on a 6-point scale—complete, good, moderate, mild, none, or worse—assessed at visits 3 and 4.
2. Difference in the change in the mean CRPS Severity Score (CSS) from the end of baseline to the end of weeks 4 and 8: CSS scores will also be divided into two sub-categories: symptoms and signs [37,38] (assessed at visits 2–4).
3. Differences in the area and intensity of mechanical allodynia, assessed by brushing a soft brush (Somedic) twice at a speed of 1–2 cm/s: the area is measured in cm and the intensity rated on an NRS (0–10) and compared to the unaffected site (assessed at visits 2–4).
4. The relationship between the primary outcome measure and the duration of CRPS (measured in months).

Tertiary outcomes are as follows:

1. Differences between groups in the Patient Global Impression of Change (PGIC), assessed at visit 4: PGIC measures the patients’ overall change from baseline on a 7-point scale [39].
2. Number of patients with a reduction of ≥30% and ≥50% reduction in the mean ADP in week 4 and week 8 compared to the baseline.
3. Mean values of ADP for all 8 weeks.
4. Differences between groups in the change in the least and worst pain in the previous 24 h, rated on an NRS from 0 to 10, assessed during the baseline week (−1) and the last week of treatment (week 8).
5. Correlations between baseline measures of CGRP nerve fiber density in skin (mm^2^) and immune cells, i.e., Langerhans cells, macrophages, and keratinocytes, inflammatory markers in serum, skin flare response (FR) (measured as a % change in blood flow from baseline over time) [40,41], and treatment outcomes as measured by the mean ADP and CSS (visits 2 and 4).
6. To measure differences from the baseline (visit 2) to visit 4 in inflammatory markers in serum, CGRP nerve fiber length density in skin (mm^2^), and immune cells, i.e., Langerhans cells, macrophages, and keratinocytes and skin FR.
7. To measure differences in passive coping and pain catastrophizing at baseline (visit 2) and at visit 4, assessed using the Pain Self-Efficacy Questionnaire (PSEQ) and Pain Catastrophizing Scale (PCS) [42,43,44].
8. Differences in symptoms of depression and anxiety, pain interference, fatigue, and sleep disturbance, assessed using the Patient-Reported Outcome Measurement Information System (PROMIS-29) short form [45,46,47,48,49,50,51] (visits 2 and 4).
9. Differences in the area and intensity of evoked pain assessed via bedside sensory testing: the pain intensity is measured at the site of maximal pain or as close as possible; cold allodynia is assessed twice using a 20 °C cold Somedic thermal roller, and the same procedure is undertaken for warm allodynia (40 °C); pinprick hyperalgesia is assessed using a pinprick stimulator as the difference in the pain score of 2 stimulations at a control site and the pain site; pain is rated on an NRS (0–10); hyperpathia is assessed via repetitive mechanical pinprick stimulation at a rate of 2 HZ for 60 s, and the pain is rated using an NRS (0–10) at 10 s intervals until the pain ceases [52] (visits 2 and 4).
10. Differences in z-scores and percentages of patients with abnormal scores on the QST, in a reduced version of the protocol by the German Network for Studying Neuropathic Pain [32] (visits 2 and 4): we will measure the CDT, WDT, CPT, HPT, DMA, MDT, MPT, and WUR at the most painful area and at a corresponding non-painful area on the contralateral limb.
11. Differences in extremity edema of the affected and unaffected limb using the gold standard water displacement method based on Archimedes’ principle [53,54] and the circumference measured in cm (visits 2 and 4).
12. Differences in skin temperature of the affected and unaffected limbs using infrared thermometer [55] (visits 2 and 4).
13. Differences in the measurement of Timed Up and Go (TUG) for lower extremities and the Nine-Hole Peg Test (9HPT) for upper extremities (measured in seconds) (visits 2 and 4).
14. The difference in the intensity of the various pain symptoms assessed using the Neuropathic Pain Symptom Inventory (NPSI) [56] (visits 2 and 4).
15. Percentages of patients with adverse effects registered in the treatment period, assessed via open-ended questions in both treatment groups.
16. Differences in pain sensitivity at baseline (visit 2) and visit 4, assessed by using the Pain Sensitivity Questionnaire (PSQ) [57].

Other outcomes are as follows:

1. Assessment of blinding: At the end of the blinded phase (visit 4), the assessment from the patient and investigator is recorded in the case report form (CRF) (whether they think the patient received an active treatment or placebo or do not know, as well as what reason this is based on (side effect, effect, or something else)).
2. Assessment of patients’ expectation to the study drug (visit 2).
3. A qualitative assessment of any difference in outcomes based on the pain intensity ratings in the pain diary and pain relief scores (visit 4).

### 2.8. Data Management Plan

In this trial, the Danish Data Protection Act will be complied with in addition to the General Data Protection Regulation (GDPR).

CRFs are completed for each subject enrolled in this study directly in REDCap (https://redcap.au.dk/), hosted at Aarhus University [28,29], to the extent possible without the use of a paper CRF. If a paper CRF is used, the data are subsequently entered into the electronic CRF in REDCap. REDCap is a secure, web-based software platform designed to support data capture for research studies, providing (1) an intuitive interface for validated data capture; (2) audit trails for tracking data manipulation and export procedures; (3) automated export procedures for seamless data downloads to common statistical packages; and (4) procedures for data integration and interoperability with external sources. Source documents include medical records, pain diaries, and CRFs. Manual personal identifiable data are kept securely locked. Data will be stored for 25 years in accordance with Good Clinical Practice (GCP) regulations. The study personnel are trained in the study procedures.

### 2.9. Sample Size Calculation

According to a previous double-blind, placebo-controlled study on intramuscular neridronate in patients with CRPS type 1, we expect an average reduction in pain intensity in patients with CRPS of 4 on an NRS (0–10) and a placebo response of 2 on the same scale. With a similar standard deviation in both groups (placebo and patients) of 2.5, a type 1 error rate of 5%, and a power of 80%, a sample size of 26 patients in each group yields a minimum detectable effect of 2 (0–10 NRS) [58]. As there are no comparable studies in CRPS, we aim for 26 patients in each group (30 to account for dropouts), as stated in the power calculation above. We will also accept 20 patients (with a power 70) and 23 patients (with a power 75) in each group, although with less power (calculation based on the same study).

### 2.10. Statistics

Baseline characteristics, including demographics and disease characteristics, will be described using means with the standard deviation (SD) or medians with the interquartile range (IQR) for continuous variables and numbers with percentages for categorial variables. We will use a flow chart to describe the inclusion of patients, including dropouts, loss to follow up, and missing data. We will also report the number of screen failures.

Statistical analysis of the primary outcome will be performed using a *t*-test (parametric) or Mann–Whitney U-test (non-parametric), where applicable. Non-dichotomous primary and secondary outcomes will be performed using a *t*-test or Mann–Whitney *U* test, where applicable. For the primary outcome, the delta values from the average pain intensity in the baseline week to the last treatment week (last 7 days) will be used. Response rates and other dichotomous data are analyzed using Fisher’s exact test or χ^2^ test. Spearman’s correlations coefficient will be used to assess predictors.

The analysis for the primary outcome is the intention-to-treat (ITT) population. The last observation carried forward (LOCF) will be the primary imputation method, and the baseline observation carried forward (BOCF) will be used as a secondary imputation method for those that do not finish the pain diary. Missing data will not be replaced. All patients who have received at least one injection will be encouraged to stay in the study, complete the diary, and come for the remaining visit after 4/8 weeks.

Significance is considered at the 5% level. If there are changes to the original statistical plan, the type of change and the date of the change will be documented, and the document will be signed by the sponsor.

### 2.11. Ethical Considerations

The trial is conducted in accordance with the Declaration of Helsinki, the ICH GCP guidelines, the GDPR, and the Danish Data Protection Act. The eligible patients receive written and oral information before informed consent is obtained. It is emphasized that participation in the study is entirely voluntary. The patients can withdraw their consent at any time for any reason without further justification.

CRPS is extremely difficult to treat, as there are only few treatment options available with limited evidence [13]. Based on insights into the mechanism behind CRPS gained from preclinical and clinical studies, we believe that fremanezumab may be effective in the treatment of CRPS. As with all medication, fremanezumab may cause adverse events, both known and unknown. If there is new information about the study medication, the patients will be informed about this so they can decide whether they want to continue in the trial or not. Any risk beyond the known adverse events is not foreseen. Fremanezumab is generally well tolerated, with few side effects [59,60]. Therefore, the side effects may be outweighed by the importance of finding a treatment for the disabling pain in CRPS. The patients can continue with their usual pain medication (with few exceptions) throughout the study, and therefore, it is unlikely that they will suffer from additional pain due to study participation.

Blood sampling and skin biopsies can cause intermittent pain and, in rare cases, hematoma, bleeding, or infection. This type of biopsy is usually well tolerated and offers minimal risk to the patients. The patients are given instructions on the aftercare of the biopsy site following the procedure. Patients can continue in the study if they do not want to have blood samples or skin biopsies taken.

All methods used in the study are clinically established, and the project manager and staff have extensive clinical experience in using them.

### 2.12. Publication

Regardless of the outcome, the results (including positive, negative, and inconclusive results) of the trial will be published in a recognized international journal. The International Committee of Medical Journal Editors (ICMJE)’s recommendations for authorship will be followed [61].

## 3. Trial Status

The inclusion of patients began in December 2023 and is expected to continue until July 2027.

## 4. Discussion

CRPS is a debilitating chronic pain condition with a profound impact on the patients’ lives. The management of CRPS is demanding. Current treatment options are poor and often fail to provide satisfactory pain relief and frequently cause significant side effects [13]. This treatment gap largely stems from an incomplete understanding of the underlying mechanisms driving pain and inflammation in CRPS in acute and chronic stages. The main aim of this study is to assess the efficacy of fremanezumab, a drug targeting CGRP, on pain in CRPS.

There are numerous strengths of this study. First, it is an investigator-initiated, randomized, double-blind, placebo-controlled trial. Second, to our knowledge, this is the first randomized, double-blind, placebo-controlled trial evaluating the effect of a CGRP monoclonal antibody for the treatment of CRPS. Hence, the results of this trial, whether positive, negative, or inconclusive, can potentially provide substantial clinical and mechanistic insights. Third, the use of an electronic pain diary will expectedly reduce recall bias and missing data for the primary outcome, as data are registered in real time. Fourth, only patients fulfilling the Budapest research criteria are included in the study, which improves the specificity of the diagnosis [27]. Fifth, patients are allowed to continue their usual pain medication, with few exceptions, in a stable dose. Sixth, a comprehensive assessment battery is undertaken to evaluate the effect of the treatment in detail. This includes multiple patient-reported outcome measures, thorough clinical assessment, and paraclinical examinations, such as blood samples and skin biopsies. Seventh, the rationale for the study is based on previous preclinical and clinical studies suggesting a key role of CGRP and neurogenic inflammation in CRPS [11,20,21,22,23,24].

However, we also acknowledge that there are several limitations of this study. CRPS is an uncommon condition, symptoms and signs can fluctuate and change permanently, and some patients experience spontaneous remission [1,2]. This could make it challenging to reach the number of patients that we aim for within the timeframe of the study. Additionally, based on mechanistic insight, we have chosen to only include patients with a disease duration of maximum 36 months and without spreading of CRPS to other extremities, which may reduce the external validity of the study. When we designed the study, oral anti-CGRP antibodies were unavailable, and it became apparent that it was not possible to make the study drug (an injectable anti-CGRP antibody) and placebo identical in appearance. Therefore, we opted for administration by unblinded third-party personnel, and participants are blindfolded during the injection. Fremanezumab was chosen as pre-filled fremanezumab syringes resemble the syringes used for the placebo as closely as possible in terms of appearance. We cannot exclude the possibility that other anti-CGRP antibodies could be more effective than fremanezumab in reducing pain in CRPS. However, the efficacy of different anti-CGRP antibodies seem to be similar in the treatment of migraine [62]. Moreover, we do not know the optimal dose of fremanezumab for CRPS. The dose of fremanezumab given in the study is the same dose as that used in the treatment of migraine. Finally, as this is a proof-of-concept study, the treatment duration is only eight weeks. Hence, we will not be able to investigate the long-term effects of treatment with anti-CGRP antibodies in CRPS.

With this study, we aim to elucidate the role of neurogenic inflammation in CRPS and to evaluate the effect of anti-CGRP antibodies on pain in CRPS. If found effective, fremanezumab and other drugs targeting CGRP may emerge as a new mechanism-based treatment option for patients with CRPS. This could hopefully improve the quality of life of patients with this devastating pain disorder.

## Figures and Tables

**Table 1:** Standard Protocol Items: Recommendations for Interventional Trials (SPIRIT) [25,26].

	Study Period						
	Screening	Baseline	Allocation	Post-Allocation			Close Out
**Timepoint**							
Week	−1		1	2	4	6	8
Day	−7 (−7 to −30)		1	14 (−4/+7)	28 (−3/+7)	42 (−4/+7)	56 (−4/+7)
**Event**	**Visit 1**		**Visit 2**	**Phone call**	**Visit 3**	**Phone call**	**Visit 4**
**Enrollment**:							
Informed consent	X						
Eligibility screen	X		X				
Demography	X						
Medical and medication histories	X		X		X		X
Pain report training	X						
Physical examination	X		X				X
Pulse and blood pressure	X		X		X		X
Pregnancy testing	X						
Randomization			X				
Concomitant medication use	X		X	X	X	X	X
**Interventions:**							
Fremanezumab			X		X		
Placebo			X		X		
**Assessment:**							
Pain diary		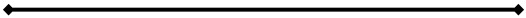					
Bedside sensory testing			X		X		X
Skin temperature	X		X		X		X
Assessment of extremity edema			X				X
Skin biopsy			X				X
Blood samples			X				X
FR			X				X
QST			X				X
CSS			X		X		X
9HPT or TUG			X				X
Pain relief					X		X
PGIC							X
PCS			X				X
NPSI			X				X
PROMIS			X				X
PSEQ			X				X
PSQ			X				X
Expectation of drug effect			X				
Adverse events			X	X	X	X	X *
Assessment of blinding							X

Schedule of enrollment, interventions, and assessment. 9HPT: Nine-Hole Peg Test; CSS: CRPS Severity Score; FR: flare response; NPSI: Neuropathic Pain Symptom Inventory; PCS: Pain Catastrophizing Scale; PGIC: Patient Global Impression of Change; PROMIS: Patient-Reported Outcome Measurement Information; PSEQ: Pain Self-Efficacy Questionnaire; PSQ: Pain Sensitivity Questionnaire; QST: quantitative sensory testing; TUG: Timed Up and Go; * If patients report adverse events at the last visit, an extra phone call is scheduled after a month.

**Table 2:** Inclusion and exclusion criteria.

**Inclusion Criteria**	
1.	Age between 18 and 75 years
2.	Confirmed CRPS (type I or II) diagnosed according to the International Association for the Study of Pain (IASP) diagnostic criteria of CRPS (Budapest criteria), with adaptation to research [27]
3.	Disease duration from 3 to 36 months
4.	Mean ADP score of at least 4 on an 11-point NRS ranging from 0 to 10, where 0 is no pain, and 10 is the worst pain imaginable during the baseline week
5.	Written informed consent
**Exclusion Criteria**	
1.	Other causes of pain and/or inflammation in the same area as CRPS or other concomitant pain/inflammation that cannot be distinguished from CRPS
2.	Spreading of CRPS to other extremities according to IASP criteria for spreading of CRPS
3.	Initiation of new medications within one month prior to inclusion, such as gabapentin, pregabalin and/or capsaicin (Qutenza), botulinum toxin type A, lidocaine patches (Versatis), TCAs or SNRIs, and corticosteroids prior to enrollment or for the duration of the randomized placebo-controlled phase of the study. Current and ongoing pain treatment will be allowed in stable doses (anticonvulsants, antidepressants, tramadol, paracetamol, and NSAIDs)
4.	Poor compliance or patients who cannot cooperate to report pain diary
5.	Unable to understand written and/or spoken Danish
6.	Severe depression, other significant psychiatric diseases, alcohol, or drug abuse
7.	Pregnancy or lactation
8.	Women of child-bearing potential (defined as any woman or adolescent who has begun menstruation) unless they use an acceptable effective contraception measure during the study and at least 6 months after or their male partner is vasectomized and their sole partner
9.	Known allergy to any component of fremanezumab
10.	Planned surgery
11.	Clinically significant and severe cardiovascular, cerebral, hepatic, or kidney disease

ADP: average daily pain; IASP: International Association for the Study of Pain; NRS: numeric rating scale; NSAID: nonsteroidal anti-inflammatory drug; SNRI: serotonin and norepinephrine reuptake inhibitor; TCA: tricyclic antidepressant.

## Data Availability

This article describes a study protocol; therefore, there are no data to share.

## Abbreviations

The following abbreviations are used in this manuscript:

9HPT	Nine-Hole Peg Test
ADP	Average daily pain
BOCF	Baseline observation carried forward
CDT	Cold detection threshold
CGRP	Calcitonin gene-related peptide
COMPACT	Core Outcome Measurement set for complex regional PAin syndrome Clinical sTudies
CPT	Cold pain threshold
CRF	Case report form
CRPS	Complex regional pain syndrome
CSS	CRPS Severity Score
CTIS	Clinical Trial Information System
DMA	Dynamic mechanical allodynia
DPRC	Danish Pain Research Center
FR	Flare response
GCP	Good Clinical Practice
GDPR	General Data Protection Regulation
HPT	Heat pain threshold
IASP	International Association for the Study of Pain
ICMJE	International Committee of Medical Journal Editors
IQR	Interquartile range
ITT	Intention to treat
LOCF	Last observation carried forward
MDT	Mechanical detection threshold
MPT	Mechanical pain threshold
NPSI	Neuropathic Pain Symptom Inventory
NRS	Numeric rating scale
PCS	Pain Catastrophizing Scale
PGIC	Patient Global Impression of Change
PROMIS	Patient-Reported Outcome Measurement Information System
PSEQ	Pain Self-Efficacy Questionnaire
PSQ	Pain Sensitivity Questionnaire
QST	Quantitative sensory testing
REDCap	Research Electronic Data Capture
SD	Standard deviation
SPIRIT	Standard Protocol Items: Recommendations for Interventional Trials
TUG	Timed Up and Go
WDT	Warm detection threshold
WUR	Wind-up ratio
